# Efficient Retrieval of Massive Ocean Remote Sensing Images via a Cloud-Based Mean-Shift Algorithm

**DOI:** 10.3390/s17071693

**Published:** 2017-07-23

**Authors:** Mengzhao Yang, Wei Song, Haibin Mei

**Affiliations:** College of Information Technology, Shanghai Ocean University, Shanghai 201306, China; mzyang@shou.edu.cn (M.Y.); wsong@shou.edu.cn (W.S.)

**Keywords:** remote sensing (RS), fast retrieval, ocean disasters, mean-shift algorithm, Hadoop system, pyramid HDFS storage

## Abstract

The rapid development of remote sensing (RS) technology has resulted in the proliferation of high-resolution images. There are challenges involved in not only storing large volumes of RS images but also in rapidly retrieving the images for ocean disaster analysis such as for storm surges and typhoon warnings. In this paper, we present an efficient retrieval of massive ocean RS images via a Cloud-based mean-shift algorithm. Distributed construction method via the pyramid model is proposed based on the maximum hierarchical layer algorithm and used to realize efficient storage structure of RS images on the Cloud platform. We achieve high-performance processing of massive RS images in the Hadoop system. Based on the pyramid Hadoop distributed file system (HDFS) storage method, an improved mean-shift algorithm for RS image retrieval is presented by fusion with the canopy algorithm via Hadoop MapReduce programming. The results show that the new method can achieve better performance for data storage than HDFS alone and WebGIS-based HDFS. Speedup and scaleup are very close to linear changes with an increase of RS images, which proves that image retrieval using our method is efficient.

## 1. Introduction

Ocean disasters have claimed thousands of lives and cause billions in economic damage each year. We must use the latest remote sensing (RS) technologies and computational methods to rapidly detect or forecast some devastating events and their effects (e.g., red and green tides, oil spills, storm surges, typhoons, and the sea ice). With the rapid development of high-resolution ocean satellites and data transmission technology, the number of RS images has grown considerably and the big data era of RS images has truly emerged. To process the massive images rapidly to meet fast requirement of ocean disaster warnings, Cloud computing platforms [1] such as MapReduce, the Hadoop system and the Spark system, need to be designed.

Efforts have been made in the past few years towards efficient storage and processing via high-performance Cloud computing technologies. Google, as one of the leading companies in the field of big data, firstly proposed the MapReduce programming model [2] which is designed to process large amounts of distributed data efficiently. Parallel processing based on MapReduce programming is used to solve some problems within the field of RS applications such as storage, detection, retrival and classification [3,4,5,6], etc. Although MapReduce is an efficient solution to the big data problem, there are a lot of limitations. MapReduce is only a computing architecture, and has no system for sharing and storing files. Hence, a distributed file system has to be combined into the MapReduce model to construct an effective Cloud computing environment. Over recent years, the Hadoop distributed file system (HDFS) has been used as an open source framework for processing and analysis of massive RS data [7], which has become a highly popular solution for storing and processing a large amount of image data for analysis purposes. In order to meet the characteristics of WebGIS accessing patterns, Liu et al. [8] proposed WebGIS-based HDFS storage to support popular web applications, but their method is sensitive to small file I/O performance, and the accessing time of their method is still relatively long. Being aware of the quality of service of the DataNodes, Cloud storage from HDFS [9,10] can increase the storage utilization ratio and improve storage performance. This makes the Hadoop distributed computing model more suitable to unstable wide area network environments. Rajak et al. [11] store the outputting results in the HBase of the Hadoop system and perform parallel RS data processing. The speedup and performance show that by utilizing Hadoop, they can distribute the workload across different clusters. In the geospatial data processing field [12], the framework is also designed to enable the storage and processing of spatial and RS data in a distributed environment.

Recently, high-resolution images have proliferated due to the advanced technology in the RS field. The size of one scene of satellite image data is 300–400 MB, and even a few GB (such as for True Marble image data, where each file is 1.5 GB). Mosaic RS images may be more than 10 GB or 50 GB [13]. If we divide large-scale RS images into too many small files such as in [11,12], HDFS has to be modified to provide better storage performance. Image pyramids are hierarchy models which are simple and effective for representing large-scale satellite images at different levels of detail. In this paper, we create a pyramid model for RS images in order to achieve image multi-resolution organization. For large-scale RS images, traditional sequential pyramid building is a time-consuming task for many fast warning requirements of ocean disasters. Hence, we build a pyramid model on the Hadoop system and realize efficient storage of large-scale ocean RS images. It has good storage and extensibility, and thus higher efficiency is achieved for massive RS images.

In the retrieval field of images, some methods via clustering algorithms have also been designed to handle images or data. Using K-means clustering techniques, approaches for retrieving semantically-relevant images [14,15] were presented. However the authors indicate there are many issues of interest to be optimized in the query stage of image retrieval. Similarly, an novel image retrieval method of an improved K-means clustering algorithm [16] was proposed and experimental results demonstrated that the proposed method can achieve a good retrieval accuracy. In order to improve retrieval speed, a fast K-means algorithm [17] was designed based on a level histogram for image retrieval and a good speedup was achieved. Later, parallel algorithms of K-means [18,19] were designed on a variety of heterogeneous computing devices to meet fast demand. On the other hand, using the modern Cloud computing platform such as the MapReduce paradigm, [20], distributed computing of fuzzy C-means (FCM) [21,22] and K-means [23,24] were investigated. Speedup of retrieval using these distribution methods is improved greatly. Despite their implementation work in correctly achieving competitive purity results compared to state-of-the art clustering algorithms, FCM and K-means have some significant limitations. They require that the number of clusters be pre-determined. In practise, it is often difficult to find the right cluster number, so we often just pick a sufficiently large cluster number. This will result in situations that some natural clusters will be represented by multiple clusters fund by the K-means algorithm. Besides, the K-means algorithm is in general incapable of finding non-convex clusters, which makes the K-means algorithm inadequate for complex non-linear data such as hyperspectral ocean RS images [25]. However, the mean-shift algorithm can be done quickly when the input data is small and implement clustering without pre-determining the cluster number. To solve the problem of massive non-linear ocean images and improve the performance bottlenecks in clustering, we adopt the technology of Cloud computing and modify the Mean-shift algorithm for distributed processing in the Hadoop system.

In order to design pyramid HDFS storage and improve scaleup for handling massive volumes of ocean typhoon images quickly, a high processing speed has become an indispensable requirement when providing fast retrieval. In this paper, we present an efficient storage and retrieval of massive typhoon image via a Cloud-based mean-shift algorithm. We design the distributed construction and tiles coding method to realize the pyramid-based HDFS storage. Higher throughput rate and construction performance are achieved. Based on the pyramid HDFS storage method, an improved mean-shift algorithm for efficient retrieval is proposed by modifying the canopy algorithm [26] to adapt to the Hadoop MapReduce framework. The specific steps of the improved mean-shift algorithm are described and significant improvements in the processing performance are obtained.

The remainder of this paper is organized as follows. The efficient storage of massive ocean image via the pyramid HDFS model is designed in Section 2. Improvement of the mean-shift algorithm via a combination with the canopy algorithm in the Hadoop system is described in Section 3. Then, experimental results are discussed in Section 4, and finally conclusions are given in Section 5.

## 2. Efficient Storage of Massive Ocean RS Images via the Pyramid HDFS Model

With a sharp increase in the size of ocean RS images (such as in typhoons), a large size (GBs or even TBs) brings a great challenge for image pyramid building. The traditional serial processing strategies of image pyramid building like the Geospatial Data Abstraction Library (GDAL) [27] take quite a long time to process large-scale ocean images, and therefore, how to quickly build pyramid for large-scale RS images becomes an urgent problem.

In this paper, we design a distributed construction method to build an efficient pyramid storage strategy of massive images on a Cloud computing platform. Firstly, we design the overall storage flow of RS images tiles including a mapping task and reducing task shown in Section 2.1, which can show the storage process of massive ocean RS images in the Hadoop system. Then, based on the overall flow chart, we can solve how to divide large-scale images into small tiles via distributed pyramid construction algorithm in Section 2.2. Later, the small tiles can be further coded and organized to two types of data structures for distributed storage in Section 2.3. Finally, original RS images are transformed to a computable data organization, and this is restored in the HDFS for processing and retrieval.

### 2.1. Storage Flow of RS Images

**Figure 1:**
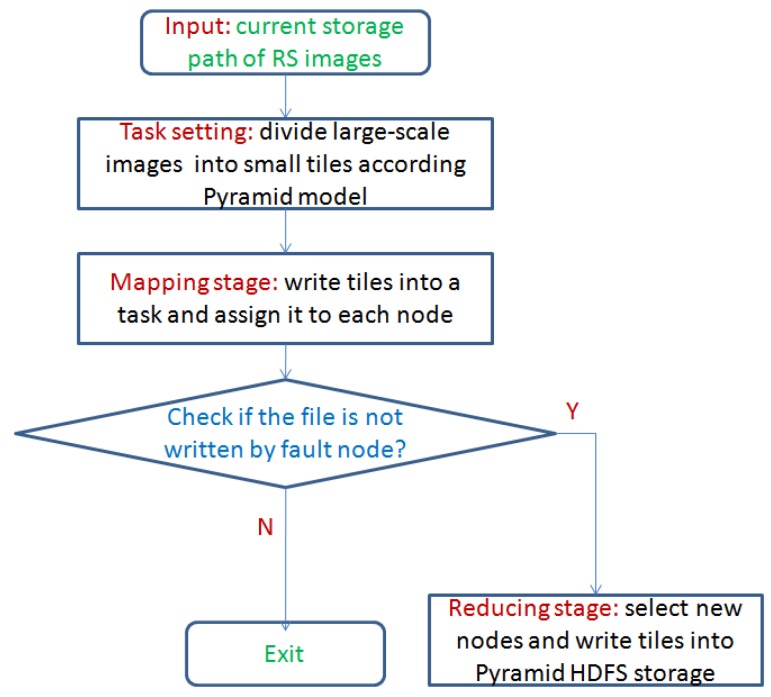
Storage flow of remote sensing (RS) images tiles.

In this paper, we design the storage flow of RS images based on the MapReduce architecture. Reading and writing tasks are divided into many smaller new tasks. Therefore we enable smaller tasks to execute independently in the distribution system, and shorten the time of the entire storage procedure. Figure 1 shows the storage flow of tile data via the pyramid HDFS model.

From Figure 1, in our design, the input RS images are divided into small tiles according to the pyramid HDFS model in the task setting stage. Then we write tiles into smaller tasks and assign them to each node in the Hadoop system. Finally, we check whether the task is completed successfully.

### 2.2. Distributed Construction Method via the Pyramid Model

Before using ocean RS images, they must be divided into tiles according to the image pyramid model. After segmentation, the size of the original image is small enough to be processed and the number of level is generally not more than 20 layers when constructing the image pyramid model. In this paper, the zoom maximum level of the RS images is influenced by the resolution of the original image. Meanwhile, considering different width and height of image sizes, the maximum number of layers needs to be calculated by two aspects: width and height. The final number of layers is selected as the maximum value from two aspects. The maximum division layer algorithm (MDLA) is described in Algorithm 1.
**Algorithm 1:** Maximum division algorithm. **Input:**
Extent is the coordinate range of original RS images; cellSize is the pixel value of RS images; tileSize is the slice size of image tile. **Output:**
level is final number of division layers.1:Input coordinate range of original RS images Extent, and evaluate the width and height of RS images: $W_{0}\leftarrow Extent.width$, $H_{0}\leftarrow Extent.height$;2:Evaluate resolution of *i*-th layer (1≤i≤20): $Res_{w}=W_{0}/\left(2^{i}\times tileSize\right)$, then i=i+1;3:If $cellSize.width+K\geq Res_{w}$ (K is a correction constant), stop evaluation and achieve the value *i* as maximum value of division layers. Or if $cellSize.width+K<Res_{w}$, repeat Step 2 to evaluate resolution of (i+1)-th layer;4:Reassign $W_{0}\leftarrow H_{0}$, and evaluate $Res_{h}$ according to processing method in Step 2 and Step 3;5:Evaluate the maximum value $level\leftarrow max(Res_{w},Res_{h})$;6:Return final number of division layers level.

Based on the maximum division layer algorithm Algorithm 1, we design the algorithm how to construct a pyramid HDFS model on the Hadoop system. The distributed construction algorithm of the image pyramid model is described in Algorithm 2.
**Algorithm 2:** Distributed construction algorithm of image pyramid model. **Input:**
RS is the source of RS images; cols and rows are number of rows and columns respectively; CellSize is a function to get pixel value; tileSize is the slice size of image tile. **Output:**
AttributeData is the attribute data; NetData is net data; 1:Read the partitioned image RS into HDFS and achieve the coordinate range of RS images Extent;2:Evaluate pixel value of RS images cellSize=CellSize(Extent.width/cols,Extent.height/rows);3:Divide layers using MDLA(Extent,tileSize,cellSize) in the Algorithm 1;4:Evaluate image resolution $Res_{i}$ when dividing each layer *i*, and produce division data divData;5:If the terminal condition of MDLA is satisfied, return final number of division layers level and then implement Step 7;6:If the terminal condition of MDLA is not satisfied, then i=i+1, and return Step 4;7:Ingest the data of current layer to HDFS with two parameters: *i*, divData;8:Based on two parameters in Step 7, call SinkLevel function to establish the pyramid HDFS model, and produce the attribute data AttributeData and net data NetData;9:If level≥i, repeat Step 8. If level<i, the repeat stops, AttributeData and NetData are outputted into the HDFS finally;

The data sets in the calculation process can be stored in HDFS, which can reduce the number of accessing data, shorten the image computation time, and improve the construction speed of the pyramid image model. Therefore, it can meet the requirements of the efficient construction of the image pyramid model and fast processing.

### 2.3. Storage Strategy of Massive RS Images

**Figure 2:**
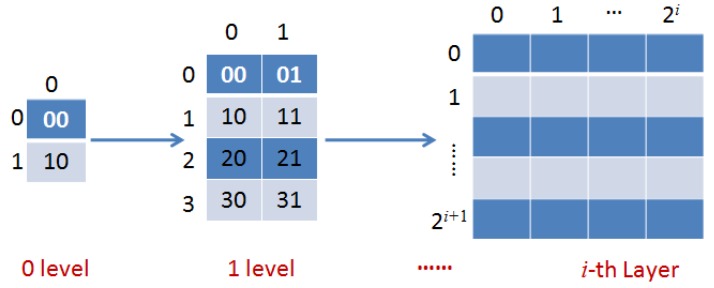
The image tiles coding process.

Traditional database storage such as ArcSDE or Oracle are quite complicated for massive ocean RS images. We use HDFS to store and manage image tiles, and do not need to build additional index information, which can greatly improve accessing efficiency of massive images. Here we mainly study an efficient storage method that is based on the distributed file system in Hadoop. After partitioning the original image into many blocks as tiles, the layer organization of the pyramid model is generated by stitching them, and the pixels in one image layer are mapped to the other image layer according to the coding process by the Tile Map Service (TMS) algorithm [28], which is the specification established by the Open Source Geospatial Foundation (OSGeo). In this paper, the tile data of each partition image is not stored separately, but firstly will be assigned to one or more DataNodes in the Hadoop system. Then the range of image tile data is located by encoding value SpatialKey and level number *i*. Finally, data values in the metadata tiles are obtained. Figure 2 shows the coding process of the pyramid structure of image tiles.

From Figure 2, the basic principle is to cut the map into tiles at the *i*-th layer, and store the tiles in the server according to the layer levels. When the map is magnified one level every time, every original 256×256 tile will be split into four 256×256 tiles. Based on TMS, we can find tiles through zoom level, column number and row number. Assuming the zoom level of images is *n* (0≤n<20), when the image is segmented based on geographic coordinate system, the column number is $2^{n+1}+1$, and row number is $2^{n}+1$.

In the coding process, due to regularity of tiles in geographic coordinate systems, we can design an accurate index relationship which is established between the encoding value and coordinates. Through the latitude and longitude of coordinates, the tile encoding value can be obtained. On the contrary, longitude interval can be also found via the encoding value. This relationship is described by the computation functions as follows.

Assuming *x* and *y* coordinates of the image tile, xtile and ytile can be achieved by the accurate index relationship as follows.

(1) $$xtile=\left(\frac{lon\_deg+180}{360}\right)\times n$$

(2) $$ytile=\frac{\left(1-\log(\tan(lat\_deg)+\sec(lat\_deg))/\pi \right)}{2}\times n$$

where lon_deg and lat_deg are the degrees of longitude and latitude, respectively.

Then the values of longitude and latitude can be derived from Equations (1) and (2) as follows.

(3) $$lon\_deg=\frac{xtile}{n}\times 360-180$$

(4) $$lat\_deg=\arctan\left(\sinh\left(\pi \times \left(1-\frac{2\times ytile}{n}\right)\right)\right)$$

Therefore, in the process of constructing pyramid model on Hadoop system, we can determine the loaded image tiles according to the central coordinates and the level number of original RS images. Similarly, when clicking on base map, we can also fix the range of latitude and longitude according to the encoding value of image. Finally the coordinates of one pixel can be exactly located on the image tile.

**Figure 3:**
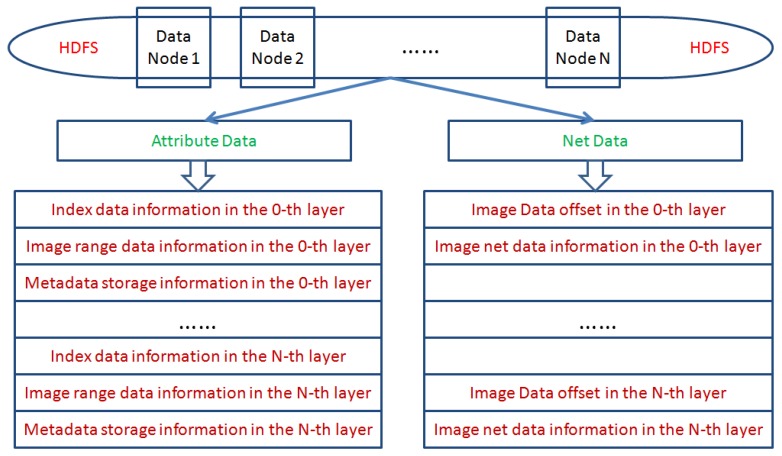
Two types of data for distributed storage. HDFS: Hadoop distributed file system.

The pyramid model in this paper is constructed based on MapReduce programming, and its purpose is to accelerate the process of image processing. We process the original RS images as a computable data organization and store it in HDFS. Two types of data structures are produced after processing. One class of data is attribute data of the JSON file format, and the other data is net data without head information as shown in Figure 3.

From Figure 3, the attribute data includes index data, image range data, and metadata, which are respectively stored in the 0-th layer to the *N*-th layer. Net data includes data offset and image net data information, which is respectively stored in the 0-th layer to the *N*-th layer. When constructing the image pyramid model, these image data will be stored in each node of HDFS.

## 3. Improvement of Mean-Shift Algorithm via Canopy Algorithm in the Hadoop System

After storing the large-scale RS images into the distributed HDFS, it is necessary to study how to use two-class data effectively in ocean disasters such as for typhoon image retrieval. We have considered the mean-shift algorithm to conduct feature clustering and image retrieval. However the traditional mean-shift algorithm can not be directly used on the Hadoop platform, and it must be improved for the Hadoop platform to adapt to the MapReduce framework. In this paper, we improve the mean-shift algorithm for large-scale RS images via combing with canopy algorithm in the the Hadoop system.

### 3.1. Iterative Mean-Shift Algorithm

Mean-shift is a nonparametric iterative algorithm or a nonparametric density gradient estimation using a generalized kernel approach, which is one of the most powerful clustering techniques.

Given *n* data points $x_{i}\left(i=1,2,3,\ldots ,n\right)$ in a *d*-dimensional space $R_{d}$, the multivariate kernel density estimate using a radially symmetric kernel (e.g., Epanechnikov and Gaussian kernels), K(x), is given by,

(5) $$f\left(x\right)=\frac{1}{nh^{d}}\sum_{i=1}^{n}K\left(\frac{x-x_{i}}{h}\right)$$

where *h* (termed the bandwidth parameter) defines the radius of kernel. The radially symmetric kernel is defined as,

(6) $$K\left(x\right)=c_{k,d}k\left({∥x∥}^{2}\right)$$

where $c_{k,d}$ represents a normalization constant which assures K(x) integrates to 1. k(x) is the profile of the kernel. The modes of the density function are located at the zeros of the gradient function ∇f(x)=0.

Then we take the gradient of the density estimator (5),

(7) $$\nabla f\left(x\right)=\frac{{2c}_{k,d}}{{nh}^{d+2}}\sum_{i=1}^{n}\left(x_{i}-x\right)g\left({∥\frac{x-x_{i}}{h}∥}^{2}\right)$$

Based on the gradient, some further algebraic manipulation yields,

(8) $$\nabla f\left(x\right)=\frac{{2c}_{k,d}}{{nh}^{d+2}}\left[\sum_{i=1}^{n}g\left({∥\frac{x-x_{i}}{h}∥}^{2}\right)\right]\left[\frac{\sum_{i=1}^{n}x_{i}g\left({∥\frac{x-x_{i}}{h}∥}^{2}\right)}{\sum_{i=1}^{n}g\left({∥\frac{x-x_{i}}{h}∥}^{2}\right)}-x\right]$$

where $g\left(x\right)=-k^{\prime }\left(x\right)$ denotes the derivative of the selected kernel profile. The first term is proportional to the density estimate at *x* (computed with the kernel $G=c_{g,d}g\left({∥x∥}^{2}\right)$). The second term, called the mean shift vector, *m*, points toward the direction of maximum increase in density and is proportional to the density gradient estimate at point *x* obtained with kernel *K*.

(9) $$\nabla m\left(x_{i}^{t}\right)=\frac{\sum_{i=1}^{n}x_{i}g\left({∥\frac{x-x_{i}}{h}∥}^{2}\right)}{\sum_{i=1}^{n}g\left({∥\frac{x-x_{i}}{h}∥}^{2}\right)}-x$$

The procedure of mean-shift for a given point $x_{i}$ is shown in three steps as follows.

1. In order to quickly find points toward the direction of maximum increase in density, evaluate the vector of mean-shift $m(x_{i}^{t})$ based on Equation (9).
2. Using $m(x_{i}^{t})$ in Step 1, translate the window of density estimation from $x_{i}$ to $x_{i+1}$: $x_{i}^{t+1}=x_{i}^{t}+m\left(x_{i}^{t}\right)$
3. Repeat Step 1 and Step 2 until convergence, i.e., $\nabla f(x_{i})=0$ according to Equation (8).

**Figure 4:**
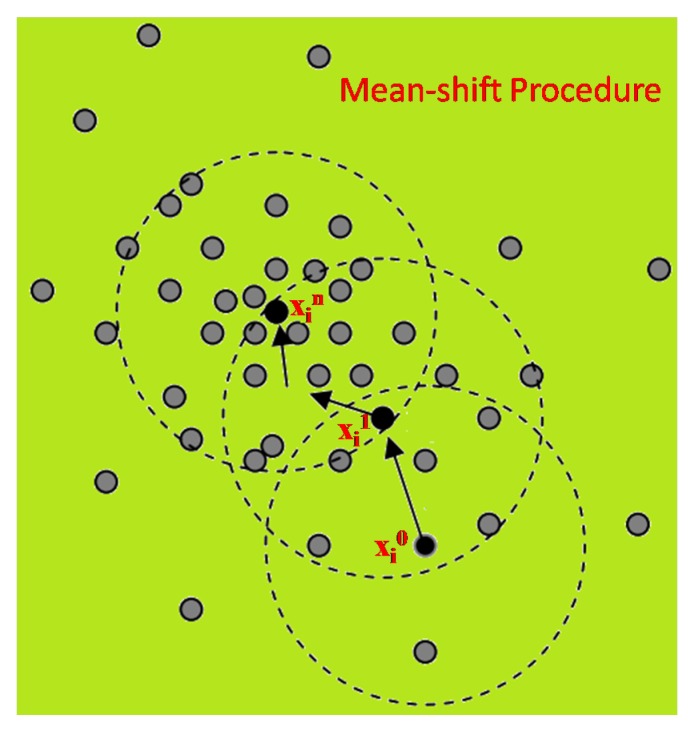
Finding procedure of mean-shift.

The mean-shift clustering algorithm can be described as a practical application of the mode finding procedure, which is illustrated in Figure 4:

From Figure 4, starting at data point $x_{i}$, the mean-shift procedure finds the stationary points of the density function. Superscripts denote the mean shift iteration, the shaded and black dots denote the input data points and successive window centres, respectively. The dotted circles denote the density estimation windows. The set of all locations that converge to the same mode defines the basin of attraction of this mode.

### 3.2. Improved Mean-Shift Algorithm via Hadoop MapReduce Programming

In order to detect or forecast ocean devastating events and their effects efficiently, special features from massive data has to be extracted rapidly and precisely. In this paper, we improve the mean-shift algorithm by fusing the canopy algorithm [29] and redesign it via Hadoop MapReduce programming. The canopy generation algorithm, called canopy clustering, is a fast and specialized algorithm to deal with massive and high-dimensional data. It divides the input data points into some overlapping clusters (called canopy). Usually, points in the canopy are reclustered with higher precision algorithm, so the canopy algorithm is often used to preprocess data, which can bring much convenience to mean-shift algorithms.

**Figure 5:**
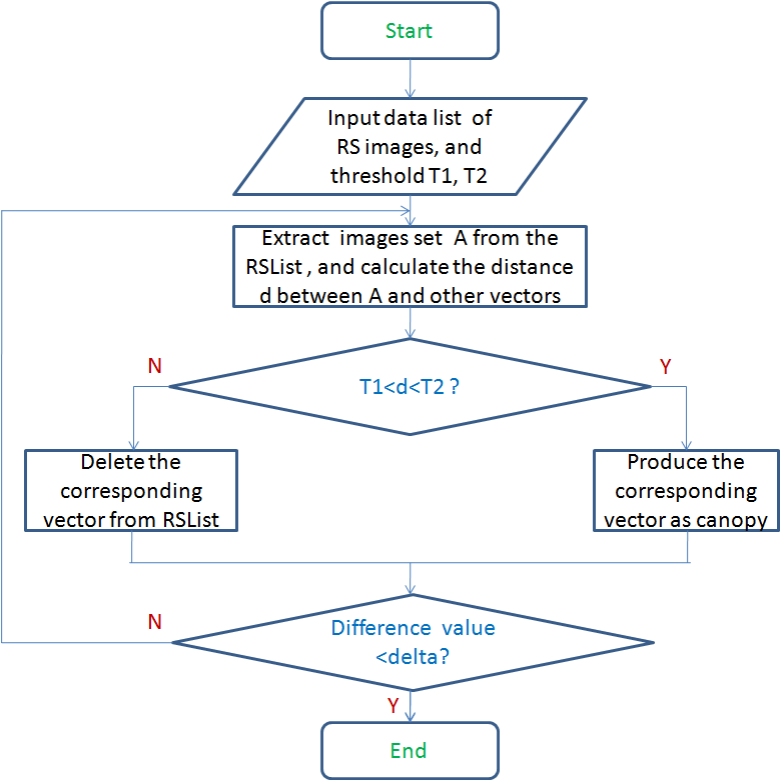
Flow of creating canopy.

In the context canopy refers to a group of similar points, or a cluster. Canopy clustering is based on two distance thresholds T1 and T2 that attempt to estimate the possible cluster centre (or canopy centre). The advantage of canopy clustering is that it gets the cluster at a very fast rate, just traversing the data to get the results. Based on the canopy clustering, we can quickly obtain the data set to estimate the number of clusters and the approximate central position. Figure 5 shows the process of creating canopy.

The canopy technique is more useful when the data set is large such as in massive ocean RS images. From Figure 5, the key idea to perform clustering is as follows.

1. The RS images RSList is firstly acquired from the storage system and sorted according to certain rules. T1 and T2 (T1<T2) are set as initial distance thresholds.
2. In order to have objectivity, select one RS image *A* from the RSList randomly. Then calculate the distance between *A* and the other vectors in the RSList using the distance calculation formula. Here, the distance calculation is obtained by Euclidean formula as follows:
  (10) $$d\left(X,Y\right)=\sqrt{\left(\sum_{i=1}^{n}{\left(x_{i}-y_{i}\right)}^{2}\right)}$$
3. Create canopy for the vector which its distance *d* acquired in Step 2 is between T1 and T2 (T1<d<T2), and remove the other vectors from the RSList table.
4. Repeat Step 2 and Step 3 until the RSList table is empty.

Based on the key idea to perform canopy clustering above, we redesign the canopy algorithm based on the MapReduce parallel computing mode. Distributed canopy clustering in the Hadoop system is implemented by tasks of Map and Reduce as follows:

1. Map task: Based on RS images RSList from HDFS in Section 2, we randomly extract a sample vector as the center vector of the canopy, and then traverse all the sample data vector and calculate the distance from the center vector. According to the distance value, some RS images are selected to create canopy. Finally the center vectors of all canopies are the output of the Map task.
2. Reduce task: Integrating the center vectors from Map task stage in Step 1, we generate a new canopy center vector as the final output.

**Figure 6:**
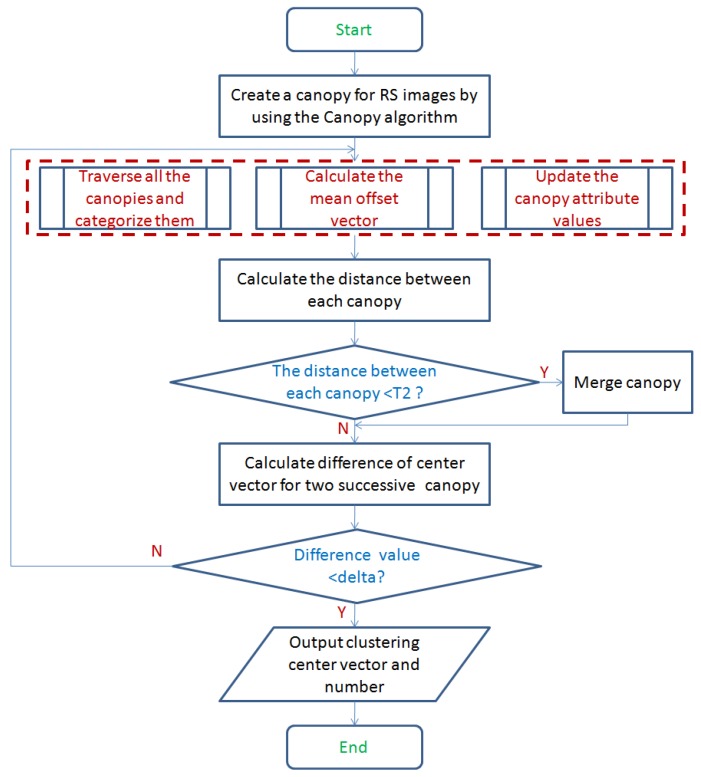
Flow diagram of mean-shift algorithm by modifying the canopy algorithm.

Before describing the improved mean-shift algorithm, we give the following parameter settings: the distance threshold T1 is set as the fixed radius of each window. The distance threshold T2 is used to determine when two canopies are merged into one. In addition, a threshold delta is set to determine whether the algorithm could stop. Figure 6 shows the flow diagram of mean-shift algorithm by modifying the canopy algorithm.

From Figure 6, the specific steps of the improved mean-shift algorithm is as follows.

1. Initially, via the canopy clustering described above, we create a canopy for each input sample data point.
2. For each canopy in Step 1, if the distance between the input value and the current canopy is less than the distance threshold T1, it would be classified as the current canopy. Each canopy computes its mean drift vector $m(x_{i})$ in Section 3.1. A new canopy center vector (the mean drift vector) can be obtained by adding the full dimension of the points contained in each canopy divided by the number of points contained. The weight of the center vector is represented by the number of points it contains.
3. For the new canopy in Step 2, we calculate the distance between them. When the distance is less than the threshold T2 (as convergence condition in Section 3.1), they would be classified as a canopy, while the corresponding attributes are also superimposed.
4. When the difference between two values of canopy which are evaluated before and after iterations is less than a given threshold delta, the algorithm ends as shown in Figure 6.
5. After the algorithm is over, the number of remaining canopy is outputted as the number of clusters.

Based on the improved mean-shift algorithm above, the distributed mean-shift algorithm fused with the canopy algorithm can be designed and implemented to adapt to the Hadoop MapReduce framework as follows.

1. In the Map phase, based on values of T1 and T2 we use the mapper function to classify the canopy created for each RS image. Then we use the new center vector to update the canopy, and finally output the results to the reducer function.
2. In the Reduce phase, based on the output results from Step 1, we use the reducer function to integrate them and then return them to driver in Step 3.
3. The driver would decide whether to continue the loop or exit by comparing the result of reducer with threshold delta.

**Figure 7:**
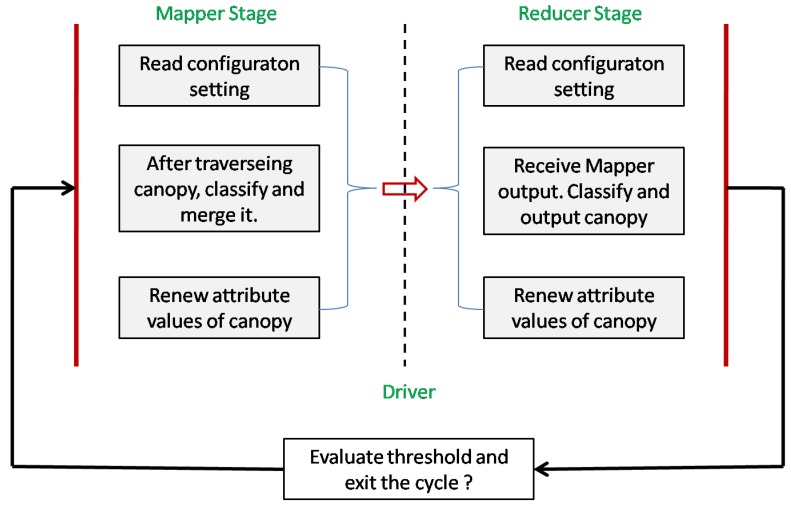
Storage structure of image data in MapReduce processing.

The main work of the algorithm’s Mapper, Reducer, and Driver can be seen in detail in Figure 7.

After clustering the significant and differential features of ocean RS images (such as rotation of typhoons in Cloud systems), the feature matching for RS images retrieval would be performed. In this paper, we use the Euclidean formula mentioned in this section to evaluate the distance difference. We calculate the distance of the RS images feature vector to be retrieved from each cluster center vector, and output the closest category scene image as the retrieval result.

## 4. Results

We have implemented the efficient storage and retrieval of massive ocean image via the Cloud-based mean-shift algorithm. The servers are running Centos OS and each has 32 GB memory. The single processor speed is 3.2 GHz using AMD Opteron 6-Core 4180. Therefore, each server has six processors and each processor has six cores. The program used for the experiments is implemented in the Scala programming language. In addition, Apache Hadoop 2.6 is used as the framework for distributing the computations on the cluster. In the experiments, the programming framework is used to provide the needed connectivity. Its component packages allow access to the MapReduce and the HDFS. All the test data is from the online satellite images of typhoons in the ocean. The images are 22 layers and 3 to 9 layers with total of 87,311 tiles are selected. The tile is large up to 164 KByte and is the smallest, 3.66 KByte.

In the test, the easy URL way is used by TMS to request typhoon image tiles, and the request method with multiple parameters is not considered here. Given an *http : //URLTyphoonLayerName/z/x/y.png*, URL is the server net address, TyphoonLayerName is the layer name of typhoon, *z* represents the zoom level of image, and (x,y) shows the image tiles coordinates.

### 4.1. Efficient Storage on Hadoop System

**Figure 8:**
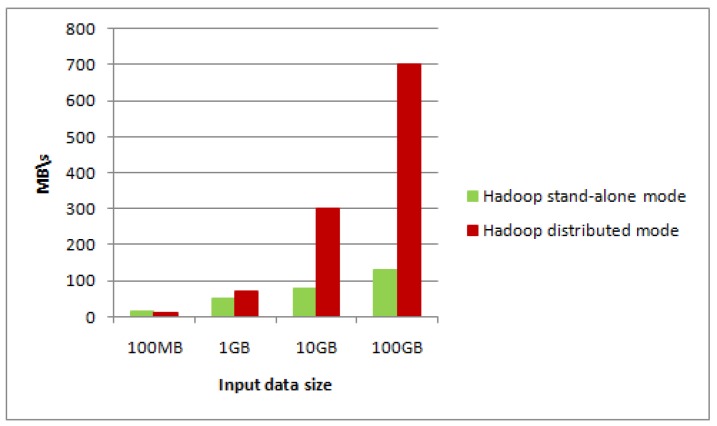
Throughput rate comparison between two modes.

**Figure 9:**
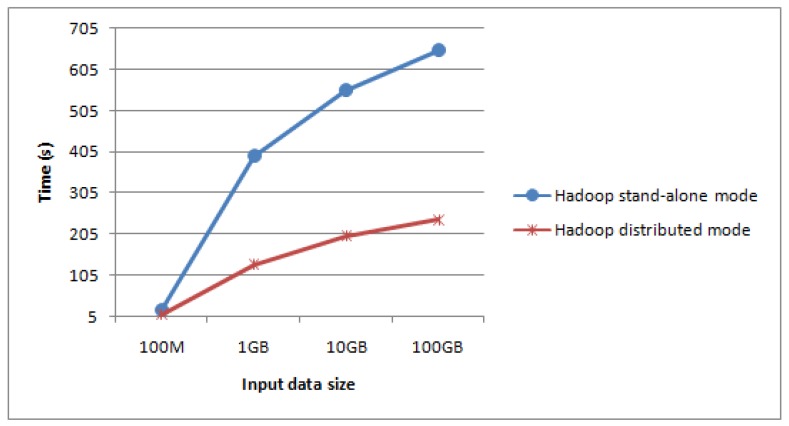
Construction performance comparison between two modes.

Firstly, the construction algorithm is respectively applied into Hadoop stand-alone mode and Hadoop distributed mode (using MapReduce programming model). Typhoon data size in the experiment is 100 MB, 1 GB, 10 GB, 100 GB, and comparison results are shown in Figure 8 and Figure 9.

From Figure 8 and Figure 9, when the data size of typhoon RS images is small, the throughput rate of stand-alone mode is higher than that of the distributed mode, however construction performance of its image pyramid is lower than that of the distributed mode. When the typhoon data size is increasing, both the throughput rate and construction performance in the pyramid distributed model are higher. In particular, when the typhoon data size of RS images is massive, performance of our method is more obvious over the stand-alone mode, which shows the achievement of our storage method.

**Figure 10:**
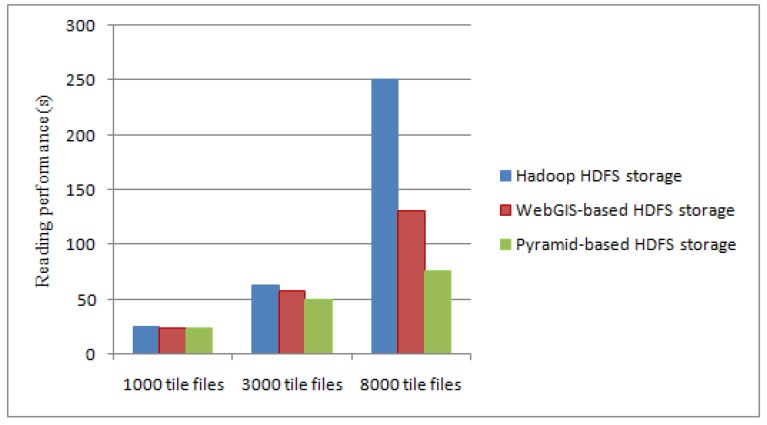
Reading time comparison of different tile data files.

In addition, we select 1000, 3000 and 8000 tiles to test the reading performance of the typhoon data. Figure 10 shows the reading time comparison of different tile data file when using traditional Hadoop HDFS storage, WebGIS-based HDFS storage [8] and our method.

From Figure 10, in the Hadoop HDFS storage, a large amount of block information is required to be read for small tile files. The reading data process is constantly jumping between many DataNodes, thus reducing the reading performance. Using the index file, WebGIS-based HDFS storage looks up the corresponding data file and reads it. It not only reduces the number of block information, but also reduces the jumping number between DataNodes when reading data. However, there is further space for improvement of the accessing time of their method. Based on the parallel framework of MapReduce, the reading data in our method is directly from each DataNode of Hadoop using a predefined pyramid storage structure. In this process, the Map task and the Reduce task take up only a little amount of time. When the amount of typhoon data is small, the performance improvement of the reading efficiency is not obvious, but with the increase in the number of files, the performance has been significantly improved.

### 4.2. Retrieval of Massive Ocean RS Images

Based on efficient storage via our method, we implement a high performance retrieval for large-scale ocean typhoon image. Test and analysis are carried out from three aspects: speedup, scaleup rate and time performance as follows.

**Figure 11:**
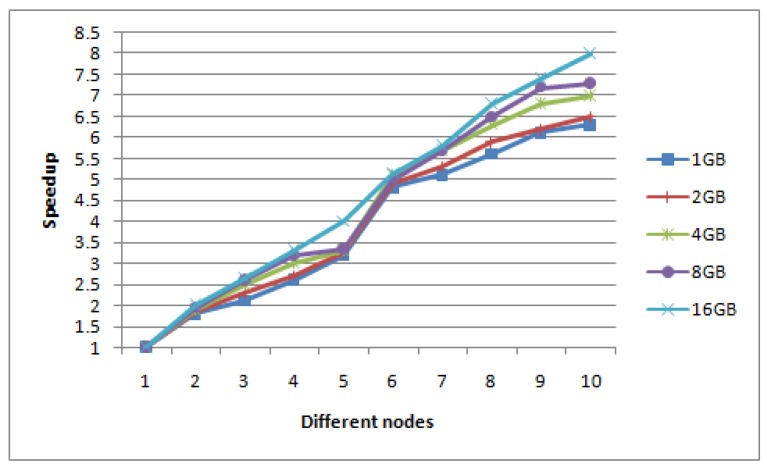
Speedup ratio in five groups of different size image sets.

The speedup is defined as the ratio of the retrieval time of Hadoop stand-alone mode to the retrieval time of Hadoop distributed mode under the same size images set. In this experiment, we firstly keep the size of the input image set unchanged, and gradually increase the number of DataNodes from one to ten to show the speedup values. Figure 11 shows the retrieval speedup ratio of five groups of different size image sets (1 G, 2 G, 4 G, 8 G, 16 G) on different DataNodes in the Hadoop distribution system.

Ideally, the speedup ratio of the system should increase linearly with the increase of the number of DataNodes, and the efficiency should always remain unchanged. But in reality, due to the I/O, communication cost and loading balance, the speedup does not increase linearly. However from Figure 11, the speedup ratio of our algorithm is close to linear. When the typhoon data set is larger, the curve is closer to linear and the speedup performance of the algorithm will get better and better.

**Figure 12:**
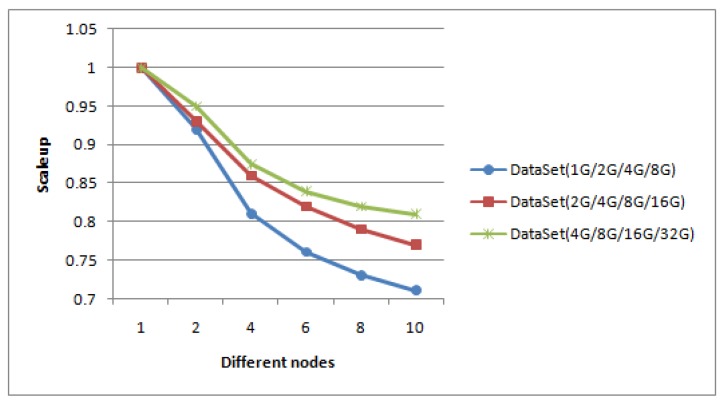
Scaleup in three group datasets.

Scalability means the ability of our method to handle a growing amount of image sets, which also refers to the capacity of distributed system to handle an increase in load. Scaleup is defined as the ratio of the computing time for processing data on one DataNode to the computing time for processing m× data on *m* DataNodes. We have performed scalability experiments with the increasing number of DataNodes, and reported the scaleup values. In our experiment, our method has good robustness and scalability. New nodes could be added into the Hadoop system at runtime to meet dynamic requirements, thus obtaining better performance in most cases and providing elastic computing as needed. Scaleup is demonstrated by using three group typhoon datasets to implement and test it. The number of data points in the datasets used are as follows: 1 G, 2 G, 4 G, 8 G in the first group, 2 G, 4 G, 8 G, 16 G in the second group, and 4 G, 8 G, 16 G, 32 G in the third group. Data in each group is implemented on 1 node, 2 nodes, 4 nodes, 6 nodes, 8 nodes and 10 nodes, respectively. Figure 12 shows the test result of scaleup performance.

From Figure 12, for the same dataset, when the number of nodes increases, the scaleup is reasonably reduced. This is because when the number of nodes increases, the communication cost between the nodes will also linearly increase. When the data size increases, the execution time of the algorithm almost linearly increase, which is in agreement with true computation and shows our method scales very well.

Also, we compare different running time for typhoon image retrieval on different sizes of the file and different DataNodes to highlight the effectiveness of our method. Firstly, the total typhoon dataset is divided in subsets with the respective sizes of the original file: 10%, 50%, 80% and 100%. Next, the data subset with 10% of original data is used for retrieval, that is, nearly 10 GB of data. Similarly, 50%, 80% and 100% of total dataset are used to implement the improved mean-shift algorithm. Table 1 shows average runtime using the improved mean-shift algorithm.

From Table 1, using half of available data it is possible to observe a improvement about 117% in the retrieval time. Even with 100% of data, the time necessary to perform a task is improved by approximately 285%, while keeping same retrieval accuracy both in training and testing. The performance would be more greatly improved with the increase sizes in the typhoon file size.

## 5. Conclusions

We have implemented an efficient storage and retrieval of massive ocean image via the Cloud-based mean-shift algorithm. From experiment and results, throughput rate and construction performance of pyramid-based HDFS storage are much higher. Especially, when the data size of RS images is massive, the advantage of our method is more obvious than the stand-alone mode. Also, our method has a shorter reading time of different tile data file over traditional Hadoop HDFS storage and WebGIS-based HDFS storage [8]. Based on the pyramid HDFS storage, the improved mean-shift algorithm for massive image retrieval is presented. Tests and analysis were carried out from three aspects: speedup, scaleup rate and time performance. The speedup ratio of our algorithm is close to linear, and our method also has good robustness and scalability. It is observed that a significant improvement in the retrieval time is achieved finally.

We have demonstrated that the proposed methods have achieved some good advantages in storage and retrieval, but many exciting directions remain to be explored. In future work, we plan on implementing some practical applications in ocean disaster warnings such as typhoon track prediction based on our method. 

## Figures and Tables

**Table 1:** Time and gain when implementing our algorithm on different sizes of the file.

Size of File	Time to Use One Node (m)	Time to Use Ten Nodes (m)	Gain (%)
10%	4.95	4.45	11.24%
50%	18.74	8.64	116.89%
80%	34.02	10.53	223.08%
100%	47.06	12.21	285.42%
